# Biophysical, Biochemical, and Molecular Docking Investigations of Anti-Glycating, Antioxidant, and Protein Structural Stability Potential of Garlic

**DOI:** 10.3390/molecules27061868

**Published:** 2022-03-14

**Authors:** Mohd W. A. Khan, Ahmed A. Otaibi, Abdulmohsen K. D. Alsukaibi, Eida M. Alshammari, Salma A. Al-Zahrani, Subuhi Sherwani, Wahid A. Khan, Ritika Saha, Smita R. Verma, Nessar Ahmed

**Affiliations:** 1 Department of Chemistry, College of Sciences, University of Ha’il, Ha’il 2440, Saudi Arabia; ahmed.alotaibi@uoh.edu.sa (A.A.O.); a.alsukaibi@uoh.edu.sa (A.K.D.A.); eida.alshammari@uoh.edu.sa (E.M.A.); s.alzahrane@uoh.edu.sa (S.A.A.-Z.); 2 Department of Biology, College of Sciences, University of Ha’il, Ha’il 2440, Saudi Arabia; s.sherwani@uoh.edu.sa; 3 Department of Clinical Biochemistry, College of Medicine, King Khalid University, Abha 61412, Saudi Arabia; wkhan@kku.edu.sa; 4 Department of Biotechnology, Delhi Technological University, Delhi 110042, India; ritikasaha_bt20a16_19@dtu.ac.in (R.S.); smitar@dtu.ac.in (S.R.V.); 5 Department of Life Sciences, Manchester Metropolitan University, Manchester M1 5GD, UK; n.ahmed@mmu.ac.uk

**Keywords:** garlic, antioxidant, anti-glycation, glycation, AGEs, HSA

## Abstract

Garlic has been reported to inhibit protein glycation, a process that underlies several disease processes, including chronic complications of diabetes mellitus. Biophysical, biochemical, and molecular docking investigations were conducted to assess anti-glycating, antioxidant, and protein structural protection activities of garlic. Results from spectral (UV and fluorescence) and circular dichroism (CD) analysis helped ascertain protein conformation and secondary structure protection against glycation to a significant extent. Further, garlic showed heat-induced protein denaturation inhibition activity (52.17%). It also inhibited glycation, advanced glycation end products (AGEs) formation as well as lent human serum albumin (HSA) protein structural stability, as revealed by reduction in browning intensity (65.23%), decrease in protein aggregation index (67.77%), and overall reduction in cross amyloid structure formation (33.26%) compared with positive controls (100%). The significant antioxidant nature of garlic was revealed by FRAP assay (58.23%) and DPPH assay (66.18%). Using molecular docking analysis, some of the important garlic metabolites were investigated for their interactions with the HSA molecule. Molecular docking analysis showed quercetin, a phenolic compound present in garlic, appears to be the most promising inhibitor of glucose interaction with the HSA molecule. Our findings show that garlic can prevent oxidative stress and glycation-induced biomolecular damage and that it can potentially be used in the treatment of several health conditions, including diabetes and other inflammatory diseases.

## 1. Introduction

AGEs are a complex and highly reactive group of heterogeneous compounds produced by glycation of proteins by reducing sugars [1,2]. Protein glycation has been shown to increase the accumulation of AGEs, the production and release of reactive oxygen species (ROS), and the structural and functional alteration of proteins, as well as resulting in tissue damage [3,4]. AGEs interact with particular receptors or bind proteins, activating a number of signaling pathways implicated in diabetic complications such as nephropathy, cataracts, Alzheimer’s disease, and atherosclerosis, among others [5,6]. Glycation, AGEs, and oxidative stress have all been linked to a variety of health problems [7]. The states of total metabolic burden, chronic hyperglycemia, oxidative stress, and inflammation have been linked to an excessive buildup of AGEs [8].

In chronic inflammation, such as that observed in rheumatoid arthritis (RA), AGEs are produced and can accumulate in tissues [9]. Besides, AGEs build with age and promote pathologic stiffening of cartilage and extracellular matrix. Pentosidine is an AGE, and it is found in blood, synovial fluid, and articular cartilage of osteoarthritis patients [9]. AGEs are linked with endothelial activation and endothelial dysfunction [10]. As a result, AGEs have been suggested as an early biomarker for cardiovascular disease [11].

Intracellular ROS inhibit glucose consumption and cause oxidative alteration of intracellular proteins. Such exposure of protein can result in fragmentation, aggregation, oxidative phosphorylation, and unwanted interactions with ion channel-coupled receptors [12]. An excessive generation of free radicals/ROS leads to oxidative stress, which is involved in the development of inflammatory illnesses such as diabetes, cancer, cardiovascular disease, Parkinson’s disease, Alzheimer’s disease, and aging. Chronic inflammation is involved in tumor development. Tissue damage and endothelial dysfunction arise from increased ROS generation at the site of inflammation, leading to inflammatory diseases [13].

Inhibition of glycation has also been shown to be beneficial in the treatment of diabetic complications [14]. Synthetic chemicals are powerful anti-glycating agents, but they can also have serious side effects such as gastrointestinal problems, uncommon vasculitis, anemia, flu-like symptoms, nausea, and diarrhea. Thus, a lot of interest has been focused on finding natural plant phytochemicals that efficiently prevent glycation and produce fewer adverse side effects. Traditional medicine practitioners frequently employ medicinal plants and natural products in their everyday practice to treat a variety of illnesses because they are generally non-toxic, cheap, ingestible, and have fewer adverse effects. [4]. The medicinal properties of garlic (*Allium sativum*) were well documented in Sanskrit literature 5000 years ago, and its use in Chinese medicine was also reported as far back as 3000 years ago. The healing properties of garlic were well known in the ancient world and utilized by the Egyptians, Babylonians, Greeks, and Romans [15]. Louis Pasteur documented the antibacterial activity of garlic in 1858. Due to its healing potential and antiseptic properties, it was used to treat and prevent gangrene during the Second World War [16]. The overall benefits of garlic in maintaining good health and in preventing a range of health issues has shifted the focus of modern-day medicine back to a time-tested natural remedy. The therapeutic roles of garlic are supported by modern-day epidemiologic evidence, with studies indicating the benefits of garlic preparations in having antioxidant and antimicrobial effects and in reducing diabetes, cardiovascular disease, and cancer [17,18]. This study focused on investigating the anti-glycation and antioxidative stress activities of garlic extract, as well as conducting an in-depth study of the secondary structural alterations of HSA proteins and how garlic extract metabolites might inhibit the glycation reactions in addition to inhibiting secondary structure alterations. This study provides a platform for the future studies in this direction, which may potentially elucidate physiological and immunological imbalances.

## 2. Results

### 2.1. Biochemical Analysis

#### Phytochemical Screening

The chemical and biological properties detected in preliminary screening of aqueous solution of garlic extract are shown in Table 1. The presence of flavonoids was determined using an alkaline reagent test. FeCl_3_ test was used to estimate phenolic content. The total phenolic compounds in garlic extract were determined to be 21.45 ± 0.02 mg gallic acid equivalent/g dry weight of the extract. Phenolic content serves as a means of protection against both infection and oxidative stress. Calculation of total flavonoid content of the extract was performed by AlCl_3_ test, with quercetin as a reference. Thus, total flavonoid content was found to be 16.58 ± 0.03 mg quercetin equivalents (QE)/g dry weight of the extract.

### 2.2. Antioxidant and Free Radical Scavenging Activities of Garlic Extract

#### 2.2.1. Assay for Ferric Reducing Antioxidant Power (FRAP)

**Figure 1:**
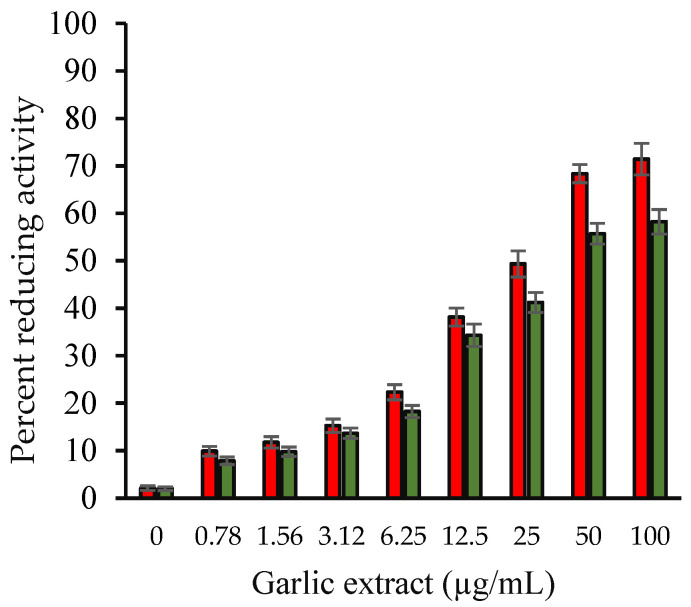
Percentage reducing power of ascorbic acid (red) and aqueous garlic extract (green). Samples in the histogram showed varying concentrations of ascorbic acid and garlic extract (0–100 µg/mL). The *y* axis shows the corresponding percentage reducing power. The results are presented as means ± SEM (*n* = 3). All the results (0.78–100 µg/mL garlic extract) were statistically significant compared with the sample without extract (0 µg/mL). Comparison between two groups was performed based on *t* test, and significance was defined as *p* < 0.05.

Using the FRAP test and ascorbic acid as a standard reference, we evaluated the reducing capability of garlic extract. The reduction of Fe^+3^ to Fe^+2^ by the extract is the basis for this test. At 700 nm, the solution of ascorbic acid (0–100 µg/mL) followed Beer’s Law with a regression coefficient (R^2^) of 0.9973 and a slope (m) of 0.004. For this figure, the intercept was 0.0216. The standard curve’s equation is y = 0.004x + 0.0216 (not given). Garlic extract had FRAP values of 32.41 ± 0.86 g ascorbic acid/100 mg dry weight of extract. Garlic extract was found to have improved ferric reducing power in a dosage-dependent manner (Figure 1).

#### 2.2.2. 2,2-Diphenyl-1-Picrylhydrazyl (DPPH) Radical Scavenging Assay

**Figure 2:**
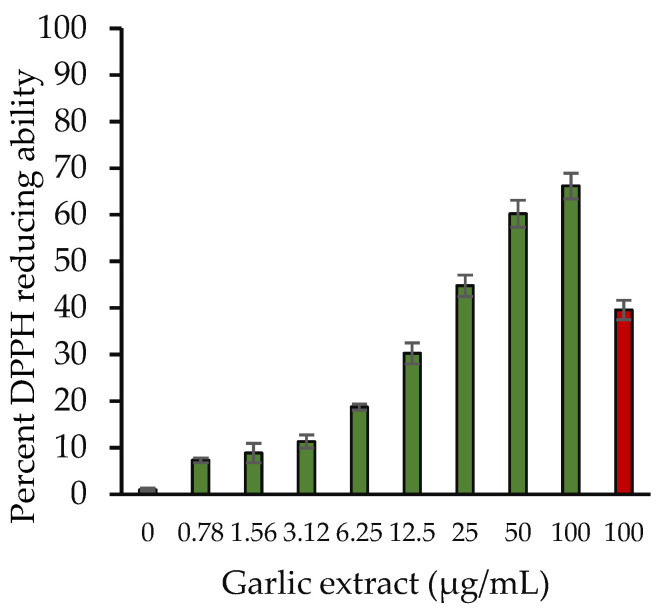
Percentage of free radical reduced vs. aqueous garlic extract concentrations. Various concentrations (0–100 µg/mL) of garlic extracts (green) and 100 µg/mL of ascorbic acid (red). The results are presented as means ± SEM (*n* = 3). All the results (0.78–100 µg/mL garlic extract) were statistically significant compared with the sample without extract (0 µg/mL). Comparison between two groups was performed based on *t* test, and significance was defined as *p* < 0.05.

The DPPH free radical scavenging method is a widely used method for determining antioxidant capacity of various compounds. Figure 2 depicts the DPPH radical scavenging capabilities of aqueous garlic extract. Garlic extract exhibits a substantial DPPH scavenging activity that was observed to rise as extract concentrations were raised from 0–100 µg/mL. The DPPH scavenging activity of 100 µg/mL extract was 66.18%. However, ascorbic acid showed 39.59% at 100 μg/mL.

### 2.3. Inhibition of Structural Changes by Garlic Extract

#### 2.3.1. Protein Denaturation Inhibition

**Figure 3:**
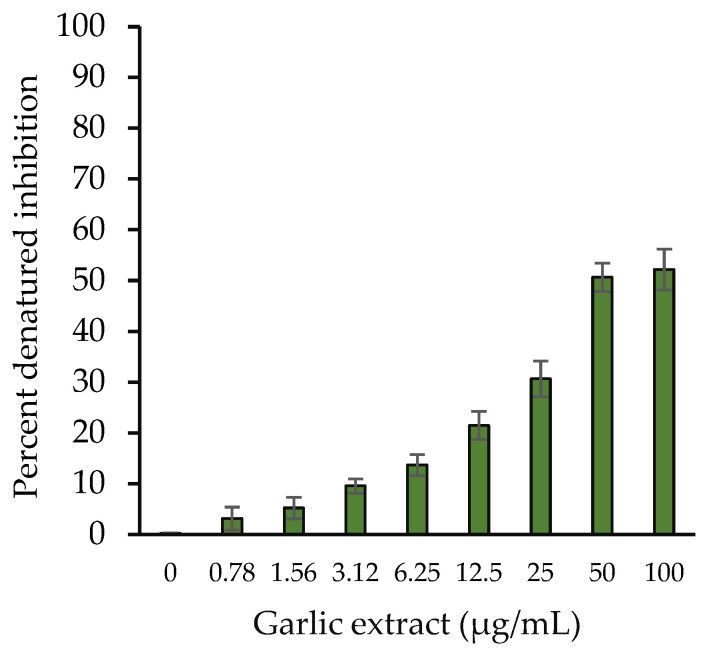
Percentage protection from denaturation induced by heat vs. garlic extract concentration (0–100 µg/mL). The results are presented as means ± SEM (*n* = 3). All the percentage denaturation inhibition results (0.78–100 µg/mL garlic extract) were statistically significant compared with the sample without extract (0 µg/mL). Comparison between two groups was performed based on *t* test, and significance was defined as *p* < 0.05.

Inhibition in in vitro HSA protein denaturation was examined using garlic extracts. Tissue proteins have been reported to be denatured by inflammatory and oxidative reactions. Natural products were used to evaluate their potential to protect proteins from denaturation and would be employed as an anti-inflammatory dietary source. Garlic extract (50 µg/mL) inhibited heat-induced albumin denaturation at a higher percentage of 50.66% (Figure 3).

#### 2.3.2. Protein Browning Inhibition

**Figure 4:**
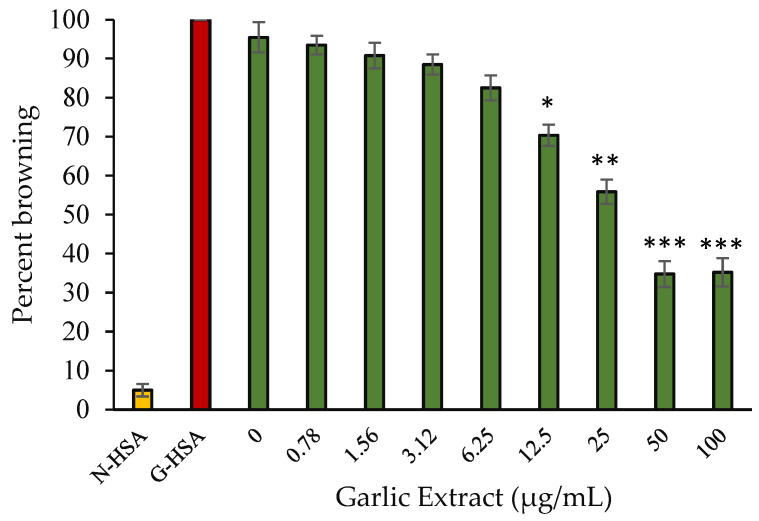
Percentage browning vs. concentration of garlic extract. Native HSA (N-HSA) and glycated HSA (G-HSA) were incubated for 10 weeks under similar conditions and are considered as negative and positive controls, respectively. G-HSA was co-incubated with garlic extract at various concentrations (0–100 µg/mL) of garlic extract (green). The results are presented as means ± SEM (*n* = 3). All the percentage browning inhibition results (0.78–100 µg/mL garlic extract) were compared with G-HSA sample. Comparison between two groups was performed based on *t* test, and significance was defined as * *p* < 0.05, ** *p* < 0.01, *** *p* < 0.001.

Browning intensity is used to assess a product’s capacity to defend against glycation. The effectiveness of extract to protect against glycation was assessed by measuring the percentage intensity of browning of HSA treated with glucose. Protein glycation causes an increase in browning intensity under hyperglycemic conditions. At 420 nm, we examined the degree of browning in samples. HSA incubated with glucose without extract had the maximum browning intensity. For this sample, we used a browning intensity of 100 percent. However, when HSA was incubated with glucose in the presence of extract, there was a noticeable reduction in browning intensity (65.23%) at a concentration of 50 µg/mL (Figure 4). In addition, in the case of HSA kept without glucose and extract, some browning (5%) was seen. Internal structural changes that occur over time might be one reason for this sample. Our hypothesis that extracts prevent the development of glycated products is supported by these findings.

#### 2.3.3. Inhibition in Protein Aggregate Formation

**Figure 5:**
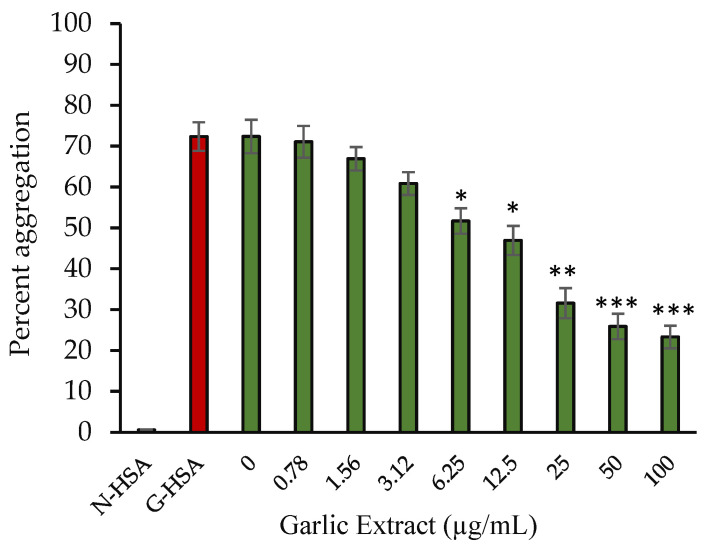
Percentage aggregation vs. concentration of garlic extract. Native HSA and G-HSA were incubated for 10 weeks and served as negative and positive controls, respectively. G-HSA was incubated with various concentrations (0–100 µg/mL) of garlic extract (green). The results are presented as means ± SEM (*n* = 3). All the percentage inhibition of protein aggregation results (0.78–100 µg/mL garlic extract) were compared with G-HSA sample. Comparison between two groups was performed based on *t* test, and significance was defined as * *p* < 0.05, ** *p* < 0.01, *** *p* < 0.001.

Glycation causes the binding of carbonyl groups to proteins, resulting in the creation of protein molecules clustering, which is also known as protein aggregation. As a result of glycation, protein aggregates formation occurs. In one of our previous studies, we showed aggregation occurs due to protein glycation [1]. Garlic extract had a beneficial effect on the inhibition of protein aggregation. The addition of extract lowered the aggregation index of G-HSA in a concentration-dependent manner (Figure 5). At a concentration of 100 µg/mL, the extract exhibited the levels of aggregation (67.77%).

#### 2.3.4. Amyloid Structure Inhibition

**Figure 6:**
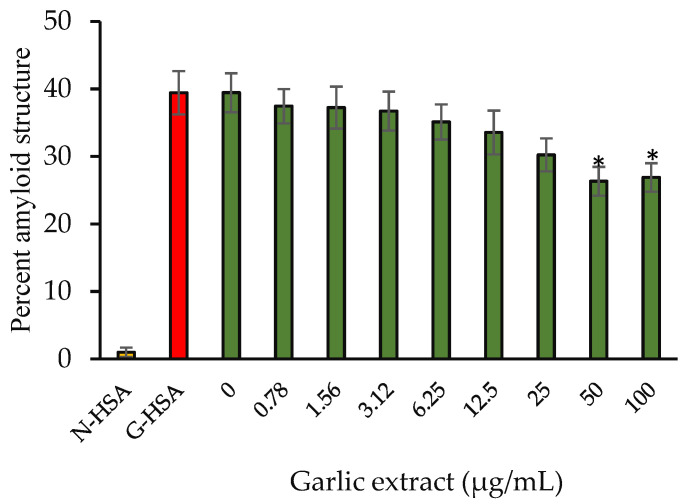
Percentage amyloid structure vs. concentration of garlic extract. Native HSA and G-HSA were incubated for 10 weeks as negative and positive controls, respectively. G-HSA was incubated with various concentrations (0–100 µg/mL) of garlic extract (green). The results are presented as means ± SEM (*n* = 3). All the percentage amyloid structure inhibition results (0.78–100 µg/mL garlic extract) were compared with G-HSA sample. Comparison between two groups was performed based on *t* test, and significance was defined as * *p* < 0.05.

Congo red (CR) dye is used to determine how much of a protein’s secondary structure has been altered. CR contacts hydrophobic clefts located in between beta fibrils and has a unique ability to bind to the protein sheet structure. After binding, the CR dye has a specific absorbance of 530 nm. In Figure 6, the findings of the CR binding experiment showed reduction in cross amyloid structure formation (33.26%) compared with positive controls (G-HSA). By masking the sites of glycation and limiting the surface area accessible by solvent, garlic extract reduced HSA fibrillation and perhaps prevented the transition from α-helix to β-sheet.

#### 2.3.5. Spectral Studies

**Figure 7:**
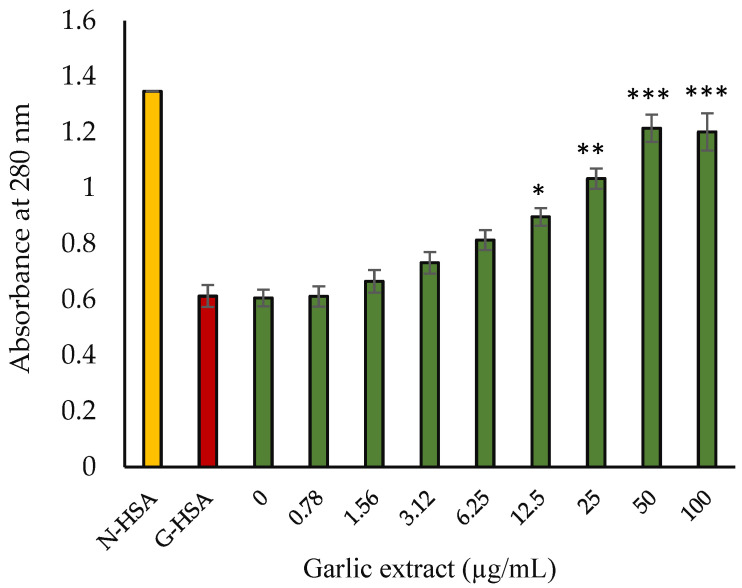
Absorbance of G-HSA vs. concentration of garlic extract. Native HSA and G-HSA were incubated for 10 weeks as negative and positive controls, respectively. G-HSA was incubated with various concentrations (0–100 µg/mL) of garlic extract (green). The results are presented as means ± SEM (*n* = 3). Change in absorbance results (0.78–100 µg/mL garlic extract) were compared with G-HSA sample. Comparison between two groups was performed based on *t* test, and significance was defined as * *p* < 0.05, ** *p* < 0.01, *** *p* < 0.001.

UV spectral studies were conducted for all the protein samples. HSA incubated with glucose has been reported to have significant decrease in absorption peak at 280 nm as compared with the native HSA, indicating considerable hypochromicity. The observed hypochromicity of glycated sample at 280 nm might be attributable to an alteration in the protein microenvironment and the change in aromatic amino acids. Glycated HSA samples incubated with the extract (50 µg/mL) showed significantly increased UV absorption (Figure 7).

**Figure 8:**
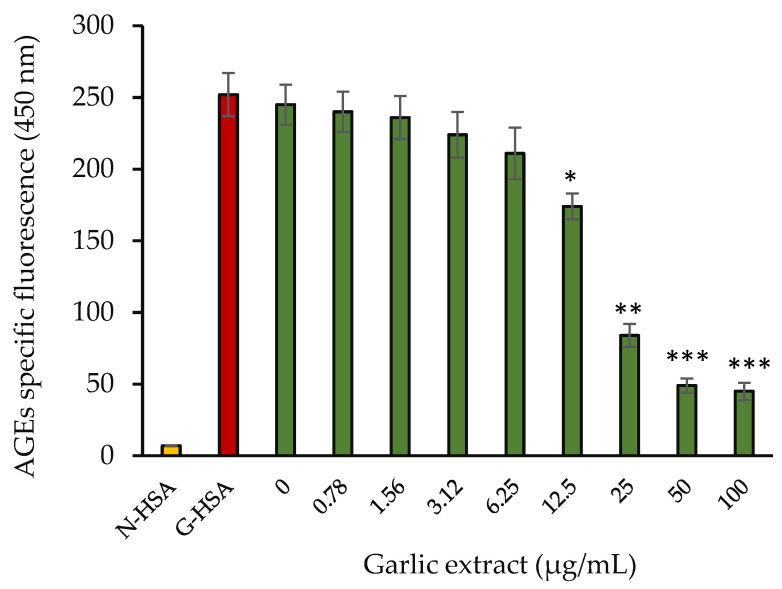
AGE-specific fluorescence intensity at 450 nm vs. concentration of garlic extract. Native HSA and G-HSA were incubated for 10 weeks as negative and positive controls, respectively. G-HSA was incubated with various concentrations (0–100 µg/mL) of garlic extract (green). The results are presented as means ± SEM (*n* = 3). Reduction in AGE fluorescence results (0.78–100 µg/mL garlic extract) were compared with G-HSA sample. Comparison between two groups was performed based on *t* test, and significance was defined as * *p* < 0.05, ** *p* < 0.01, *** *p* < 0.001.

The formation of AGEs in the glycated samples was detected using AGE pentosidine-specific autofluorescence. For each sample, the specific fluorescence of AGEs was observed to be in the range of 400–480 nm. The fluorescence intensity vs. wavelength (400–480 nm) spectra were found to be rather wide (data not shown), which might be attributable to the variety of fluorescent molecules created during the glycation process. Extract containing samples showed a steady reduction in AGE-specific fluorescence at 450 nm as the extract concentration increased in the incubated samples (Figure 8). The fluorescence intensity of HSA treated with glucose was maximum at 450 nm. Our data show that garlic extract exhibited protection against AGEs synthesis by glycation. Protection level increased as the concentration of garlic extract increased.

#### 2.3.6. Circular Dichroism

Analysis of secondary structure change with glycating agent ‘glucose’ alone or together with garlic extract with varying concentrations (0–100 µg/mL) was investigated using a Jasco J 810 spectropolarimeter (Table 2). The presence of secondary structural elements was estimated as relative percentages by using the Chen and Yang equation [19] via a computer data processor. CD spectra were recorded for all the samples at a similar wavelength range (200 to 280 nm). As we have shown previously, there is a significant change in α-helix (−10.5%) and β-sheet (+14.9%) structures upon glycation of HSA [1]. Inhibition in the change of both α-helix (−2.8%) and β-sheet (+4.6%) structures of glycated samples were observed when incubated with garlic extract of 100 (μg/mL). Significant decreases in all the secondary structure (α-helix (*p* < 0.05), β-sheet (*p* < 0.01), β-turns (*p* < 0.01), and random coils (*p* < 0.05)) changes in glycated HSA samples were observed at 25 µg/mL concentration of garlic extract. Furthermore, the highest reduction in all the secondary structures was achieved at 100 µg/mL concentration of extract used (Table 2).

### 2.4. Molecular Docking Studies for Potential Natural Product Metabolites as Inhibitors of Glycation

**Figure 9:**
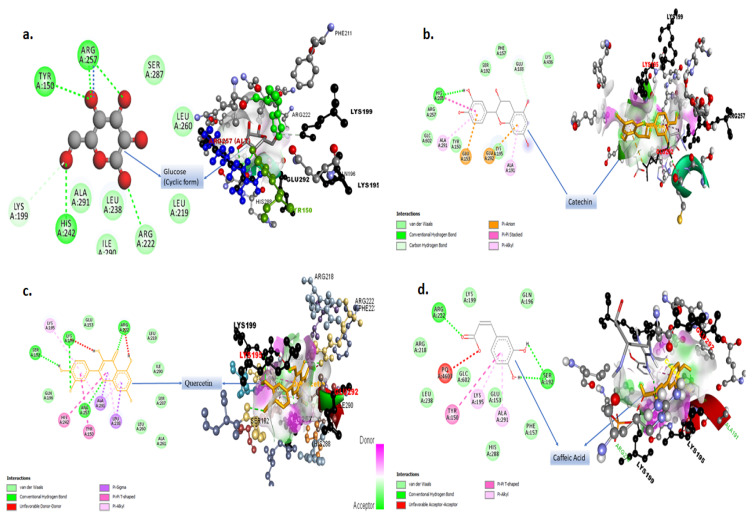
2D and 3D interaction models of ligands with HSA. Ligands used are (**a**) glucose, (**b**) catechin, (**c**) quercetin, and (**d**) caffeic acid.

**Figure 10:**
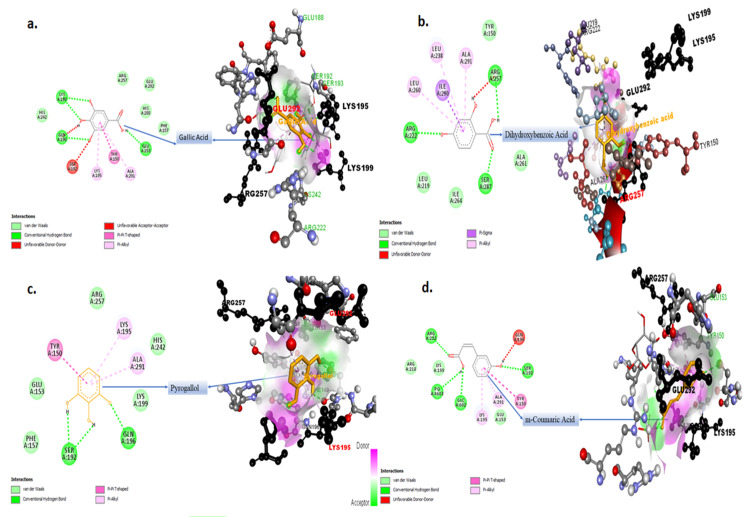
2D and 3D interaction models of ligands with HSA. Ligands used are (**a**) gallic acid, (**b**) dihydroxy benzoic acid, (**c**) pyrogallol, and (**d**) m-coumaric acid.

Some of the important phenolic compounds present in garlic extracts were analyzed for their inhibitory role in the glycation reaction. Sudlow Site I is the primary binding site for glucose in pyranose form. The interaction of the glucose ring form and other ligands (considered in this study) with the SAL subsite of the Sudlow Site I are summarized in Table 1. Figure 9 and Figure 10 provide insight into the most stable 2D and 3D docked conformations of the ligands at the SAL subsite obtained via exhaustive molecular docking application. The orientation of the glucopyranose at the SAL subsite was obtained from the crystal of glucose-bound has submitted by Wang et. al. [20] to the protein data bank (PDB ID: 4iw2). The binding efficiency reported in Table 3 is a result of multiple interactions of the ligands with the target active site.

The blue dotted interaction of Arg257 with glucopyranose (Figure 9a) represents an altered confirmation state of glucopyranose. The 2D interaction images in Figure 9 and Figure 10 represent the type and count of interactions specific ligand undergoes. The 3D images of docked ligands in Figure 9 and Figure 10 represent the polarity distribution at the docking site. The pink environment in the close vicinity of the ligands represents hydrogen donors or an electronegative environment; however, the green cloud near the ligands is due to amino acid with a side chain rich in hydrogen acceptors and results in an electropositive environment. The white cloudy areas around the ligands constitute the neutral space due to the presence of hydrophobic amino acids.

## 3. Discussion

The core cause of many lethal diseases is thought to be connected to metabolic disturbances and inflammatory changes. Diabetes mellitus, characterized by hyperglycemia, is a severe health concern that affects people all over the world [21]. Long-term hyperglycemia has been linked to diabetes complications and biomolecule glycation [22,23,24].

Nutrition looks at how people might use their food choices to reduce their disease risk and manage their illnesses. If a person’s diet lacks the right nutritional balance, they are more prone to develop a variety of health problems. When a person consumes excess or very little amounts of a nutrient, it might cause sickness. A balanced diet, according to growing data, may help you avoid problems including heart disease, cancer, osteoporosis, and type 2 diabetes [25].

Garlic has been reported to have potential therapeutic properties due to its biochemical constituents. Garlic extract can be used against glycation and AGE-related health complications linked with chronic diseases in diabetic patients due to its broad therapeutic potential [26,27]. Compounds such as quercetin, pyrogallol, caffeic acid, gallic acid, m-coumaric acid, and their derivatives are among the beneficial chemicals found in garlics. A high quantity of quercetin has been found in garlic, which is a potent antioxidant compound [28]. The antioxidant capabilities of garlic were well studied in the present investigation through various in vitro methods.

Biochemical analysis of this study showed the presence of phenolic compounds and flavonoids in a respectable amount in garlic extract [28]. According to the current findings, methanolic extract of garlic has a considerable potential to scavenge free radicals, as estimated using DPPH. Garlic also showed high reducing activity that increased with higher concentrations of the extract. It is generally known that the breakdown of H_2_O_2_ produces hydroxyl free radicals in the blood. The strong antioxidant properties are attributed to the polyphenolic compounds and polysaccharides. The polysaccharides and polyphenolic compounds in garlic might prevent free radical formation, making it an effective antioxidant.

Lowering chronic inflammation may help to postpone, prevent, and possibly treat a variety of chronic illnesses, including cancer [29]. Besides, many ROS, free radicals including NO, superoxide (O_2_^–^), and their reaction product peroxynitrite (ONOO^–^) are produced in excessive quantities during the host’s response to infections and inflammatory circumstances [16]. Furthermore, oxidative stress and inflammation are connected in glycation pathways. Identifying alternatives to non-steroidal anti-inflammatory drugs and developing innovative, effective, and safe anti-inflammatory medicines has long been a key priority. External stress as well as several compounds may cause protein denaturation that leads to the loss of structural integrity of proteins and thus loss of their functions [30,31]. Our results suggest that garlic extract can inhibit the heat-induced denaturation of HSA.

Several diseases such as familial amyloidosis, Alzheimer’s, pancreatic islet amyloidosis, etc. are caused by protein aggregation in the circulation and in organs [32]. These aggregates undergo further reactions and form amyloid fibrils that contain cross beta structures. In glycation reactions, reducing carbohydrates non-enzymatically and covalently binds to lysine and arginine groups of proteins as well as with the N terminus of polypeptides [33]. Increased levels of these aggregates may cause neurological degeneration. It has been evident that protein glycation can induce aggregation. Intense browning was observed in the polyacrylamide gel electrophoresis glycated samples of albumin [1,33].

Glycation-induced microenvironment structural alterations were observed through spectral studies that included UV spectra and AGE-specific fluorescence. These alterations were inhibited when garlic extract was present in the reaction mixture during the incubation of the glycation reaction. However, a significant inhibition in microenvironment alterations was observed at higher concentrations of the extract. Consequently, extracts lent protection from structural alterations to the protein.

Further in-depth analysis of structural alterations in glycated proteins, including the inhibitory effect of garlic extract on these changes, was investigated using CD. CD analysis of glycated HSA showed protein destabilization and reduction in α-helix structure [34]. It has been reported that changes in CD spectra of glycated HSA are based on glucose concentration. It has also been observed that upon HSA glycation there was a partial denaturation with alterations in structural integrity at different glucose concentrations (1 mg/mL and 5 mg/mL) [35]. Some authors showed that glycated albumin was more favored in a β-sheet conformation structure [36]. These previous studies showed coherence with our CD results of native and glycated HSA. Moreover, CD experimental analysis revealed that garlic extract provided protection of protein secondary structure alterations and inhibited the conversion of α-helix to β-pleated sheet structure. There are possibilities of the interaction of the metabolites or compounds present in garlic extract (Table 3) with the glycation sites on the HSA molecule, causing inhibition in secondary structure alterations (conversion of a-helix to β-sheet). These inhibitions in structural alteration of glycated HSA are highly important for their functional integrity of the protein.

Sudlow Site I is made up of three subsites: the SAL subsite, deep-seated at the bottom of the Sudlow Site I and comprised of hydrophobic residues Leu-238 and Ala-291 and the indomethacin (IMD) and 3`-azido-3`-deoxythymidine (ADT) subsites situated near the opening of the Sudlow Site I are rich in positively charged residues, Arg-218, Lys-195, and Glu-292 [20]. In blood plasma, the D-glucose found in blood plasma is a mixture of two anomers—i.e., α-D-glucopyranose and β-D glucopyranose [37]. The mutarotation between the open aldehyde chain form and ring form is quick and dependent upon medium conditions. Thus, glucose can potentially react, as an open aldehyde form or closed ring form, within a short time frame [37]. Wang Yu et al. reported the mechanism of glucose interaction with HSA [20]. Two molecules of glucopyranose were found to interact subsequently at the Sudlow Site I [20]. The first molecule gets bottom deep-seated at the SAL subsite, and this configuration is stabilized by the hydrophobic interactions involving Leu238 and Ala291 (Figure 9a). The second glucopyranose molecule is held at the entrance of the Sudlow Site I marked by IMD and AZT subsites. This region is rich in positively charged residues Lys-195, Arg-218, and Glu-292. The oxygen atom bound to C5 (carbon 5 of the second glucopyranose molecule) is attacked (protonated) by the Lys199. This releases C1 atom, which undergoes a covalent interaction with the Lys195, providing strong stability to the second glucopyranose molecule bound to HSA. The presence of a stable ligand at the SAL subsite of the Sudlow Site I reduces the propensity of protonation of the second glucopyranose molecule by the Lys199. It can be observed that quercetin and gallic acid undergoes a conventional hydrogen bonding interaction with Lys199. In both cases the Lys199 serves as a hydrogen donor; hence, this hydrogen is not available for protonation of C5 of the second glucopyranose molecule. Therefore, no covalent interaction could be established between the second glucopyranose and Lys195. This makes the second glucose molecule, at the entrance of Sudlow Site I, more vulnerable for replacement by a more stable competitor. Since quercetin, gallic acid, catechin, and caffeic acid offer more stable interaction as compared with a non-covalently bound glucose molecule (Table 3), they can offer strong competition.

Quercetin appears to be the most promising inhibitor of glucose interaction since it efficiently prevents the formation of a covalent bond by directly interacting with the Lys199. Interaction of quercetin with the SAL subsite is stabilized by the hydrophobic interaction with Ala291 and Leu238—the same amino acids involved in the stabilization of the first glucopyranose molecule at the SAL subsite. The 3-4 dihydroxyphenyl ring bound to C2 of the deep seated 3,5,7-trihydroxychromen-4-one of quercetin at the SAL subsite (Figure 9c) extends well beyond the Lys199 wall, as described by Wang et al. [20]. This sterically hinders the settlement of the second glucopyranose molecule at the opening of the Sudlow Site I, hence inhibiting the glucose interaction. Gallic acid also prevents covalent bond formation (even more efficiently than quercetin due to two possible hydrogen bond interaction); however, due to the small size of trihydroxy benzoic acid, it remains deep-seated at the SAL subsite and well within the wall defined by the Lys199, thereby incapable of preventing the entry of the second glucopyranose molecule at Sudlow Site I.

Uncontrolled levels of blood glucose together with oxidative stress create conditions with high possibilities for the formation of intermediary metabolites of glycation and AGEs. These AGEs and AGE-related metabolites exert structural and functional alterations of blood proteins, contributing to further complications in patients with hyperglycemia as well patients with other inflammatory diseases. In our study, we proved that the natural products that are present in garlic extract have antioxidant and anti-glycation properties and lend protection against the structural destabilization of proteins such as HSA. As a result, this research will help researchers better grasp the link between glycation and natural products that could be beneficial for disease prevention mechanisms in humans.

## 4. Materials and Methods

### 4.1. Materials

Ascorbic acid, trichloroacetic acid, DPPH, Folin–Ciocalteau reagent, ferric chloride, potassium ferricyanide, gallic acid, trypsin, quercetin, and Congo red were purchased from Sigma (Saint Louis, MO, USA). Hydrochloric acid, aluminum chloride, mono sodium dihydrogen phosphate, DMSO, ethanol, methanol, disodium hydrogen phosphate, sodium hydroxide, sodium carbonate, and hydrogen peroxide were purchased from Merck (Darmstadt, Germany). All chemicals and reagents were of the highest analytical grade. All solvents used were HPLC grade.

### 4.2. Chemical and Biological Studies

#### 4.2.1. Preparation of Aqueous Solution of Garlic Extract

Soft-necked varieties of garlic, i.e., *Allium sativum* var *sativum* were obtained from the local markets in Hail, KSA. Fresh aqueous extract was prepared using a previously published method with slight modifications [26]. The concentration of the aqueous solution was determined based on the total garlic used and the final volume of the extract (100 mL of aqueous extract contained 50 g of garlic, i.e., 500 mg/mL).

#### 4.2.2. Qualitative and Quantitative Analysis for Flavonoids and Phenolics

Qualitative analysis: The presence of flavonoids (alkaline reagent test) and phenolic (FeCl_3_ test) compounds in the aqueous garlic extract was checked using a protocol published by Alsahli et al. with slight modifications [38].

Quantitative analysis of total phenol and flavonoids content: Total phenol content was estimated using Folin– Ciocalteau reagent as described previously with slight modifications [26]. Gallic acid (0–100 μg/mL) served as a reference point. The total phenolic content in garlic extract was determined from the calibration plot and expressed as mg gallic acid equivalents (GAE). All assays were performed in triplicate. Results are expressed as mg gallic acid equivalent per g dry extract.

Total phenolic content (TPC) = C × V/M

where C is the concentration of gallic acid in mg/mL that was obtained from gallic acid calibration curve, V is the volume of plant extract in mL, and M is the weight of pure plant extract in grams (g).

The aluminum chloride (AlCl_3_) colorimetric method was used to determine total flavonoid content in garlic extract as described previously [12,39]. The calibration curve was plotted using quercetin (0–250 µg/mL). Garlic extract (50 µg/mL) or standard quercetin solution was added to 2% AlCl_3_ (500 µL). The resultant solution was incubated for 1 h with occasional stirring. The absorbance of reaction mixture was estimated using a spectrophotometer at 430 nm using ethanol as blank, as 2% AlCl_3_ solution was prepared in ethanol. Total flavonoid content was determined to be quercetin equivalent (mg/g) (mg QUE/g).

Total flavonoid content (TFC) = Z × V/m

where Z is concentration of quercetin (mg/mL); V is volume (mL) of sample used in the extraction; m is weight of pure dried sample used (g).

#### 4.2.3. Antioxidant and Free Radical Scavenging Activities of Extract

Antioxidant activity—reducing power: The ferric reducing antioxidant power (FRAP) technique was used to measure in vitro antioxidant activity [37]. An amount of 1 mL ascorbic acid (0–100 µg/mL) or garlic extract (0–100 µg/mL) was combined with 2.5 mL in phosphate buffer (0.1 M, pH 6.6) and 1% potassium ferricyanide (2.5 mL). After 20 min at 50 °C, 2.5 mL of trichloroacetic acid (10%) was added to the test tubes to terminate reaction. The mixtures were subsequently centrifuged at 3000 rpm for 10 min, causing the formation of supernatant. Finally, freshly made ferric chloride (0.5 mL, 0.5%) solution was mixed with the supernatant (2.5 mL) and distilled water mixture (2.5 mL). The absorbances of various samples were measured at 700 nm.

Percentage free radical scavenging activity = [(X_control_ – X_sample_)/X_control_] × 100

X_control_ = absorbance of control sample.

X_sample_ = absorbance of sample in the presence of extract.

Analysis of free radical scavenging activity by DPPH method: Antioxidant activity of aqueous garlic extracts was estimated using 1,1 difenyl-2-picryl-hydrazyl (DPPH) as published before [12]. Dry powder was obtained from the aqueous extract of garlic. This was dissolved in methanol. One milliliter of 0.3 mM DPPH in methanol was added to the extract solution test sample (2.5 mL) of varying concentrations (0–100 µg/mL) and incubated in the dark for 30 min at room temperature. All samples were read at 517 nm with methanol used as a blank. Inhibitory effect of DPPH was calculated according to the following equation:

Percentage of free radical scavenging activity = [(Ac − As)/Ac] × 100

where, Ac = absorbance of control, and As = absorbance in presence of extract.

### 4.3. Modification of HSA by Glucose

HSA glycation was carried out as described previously with minor changes [1,2]. Solutions of HSA (3 mg/mL) with and without glucose were incubated for 10 weeks under identical experimental conditions. To analyze the anti-glycation activity of garlic extract, HSA with glucose was also incubated with varying concentrations of garlic extract (0–100 µg/mL) and incubated under similar conditions. Post-incubation, all samples were dialyzed against PBS. Samples were stored at −20 °C. Protein concentrations were determined by NanoDrop™ 2000/2000c spectrophotometer (Thermo Scientific, USA).

### 4.4. Protein Denaturation Inhibition by Garlic Extract

The protein denaturation inhibition function of garlic extract was studied as described by Sakat et al. [40] and Pandey et al. [41]. A portion of 1% aqueous solution (500 μL) of HSA was added separately to 100 μL of varying concentrations (0–100 µg/mL) of garlic extract. First, the mixtures were incubated at 37 °C for 20 min and then heated for 20 min at 51 °C. Subsequently, the samples were cooled, and turbidity was measured at 660 nm. All samples were run in triplicate.

The percentage inhibition of protein denaturation was calculated using the following equation:

Percentage Inhibition = [(Ac − As)/Ac] × 100

where Ac = absorbance of control, and As = absorbance in presence of extract.

### 4.5. Measurement of Browning in Glycated Samples

The glycation process is a non-enzymatic event that can produce HSA denaturation. Glycation promotes the browning of proteins by glucose in diabetes. As a result, browning intensity can be used as a preliminary screen for glycation. The intensity of browning of glycated materials was measured by absorbance at 420 nm using a 1 cm path length cell after dilution with distilled water [12]. Experiments were performed in triplicate.

Percentage protection from browning = [(A_control_ – A_sample_)/A_control_] × 100

where, A_control_ = absorbance of HSA and glucose system, and A_sample_ = absorbance of HSA and glucose system incubated with extract.

### 4.6. Effect of Garlic Extract on Protein Aggregation Index

Glycation is a major driver of biomolecular structural changes, particularly proteins, resulting in the development of aggregates [42]. Garlic extract was tested for its ability to protect against protein aggregation caused by glycation by determining glycated sample absorbance, treated with glucose, either in the absence or presence of extract (0.78–100 µg/mL). Aggregation index was determined for each sample using their absorbance at 340 and 280 nm [12].

Percentage of protein aggregation index = [A_340_/(A_280_ − A_340_)] × 100

where, A_340_ = absorbance at 340 nm, and A_280_ = absorbance at 280 nm.

### 4.7. Spectral Studies

UV absorption measurements were taken with a Perkin Elmer Lambda 35 spectrophotometer (Waltham, MA, USA) with two beams. In the absence/presence of glucose and garlic extract, the UV spectra of HSA (0.2 mg/mL) were measured at a wavelength range of 240–500 nm. Each sample’s absorbance intensity was measured at 280 nm [2,42].

AGE-specific fluorescence study: Fluorescence measurements were performed with a Shimadzu spectrofluorometer (model RF-5301PC, Tokyo, Japan). Excitation (350 nm) and emission (400–480 nm) wavelengths were used to determine the generation of fluorescent AGE products. Slit width of 3 nm was used for both excitation and emission [38].

### 4.8. Circular Dichroism

The CD of native and glycated samples of HSA (2.2 μM), either with or without garlic extracts (0.78–100 µg/mL), was recorded on a Jasco J 810 spectropolarimeter (Tokyo, Japan). The spectropolarimeter has a temperature-controlled sample cell holder attached to a NESLAB model RYE 110 water bath (Tokyo, Japan). [1]. Path length of cuvettes used was 1–10 mm. Each spectrum was the average of three scans. CD spectra were recorded over a wavelength range of 200 to 280 nm and sensitivity of at 5 mm/millidegree (mdeg). Sodium phosphate buffer (20 mM, pH 7.4) was used to prepare all protein solutions. Presence of secondary structural elements was estimated as relative percentage by Chen and Yang equation [19].

### 4.9. Structural Details of Potential Natural Product Ligands/Inhibitors of HSA Glycation

Natural products contain several phenolic compounds and their metabolites that might be involved in inhibiting the glycation reactions. Some of the competing ligands/inhibitors are given in Table 3, with the HSA molecule characterized using molecular docking studies. All the ligands’ .sdf files were derived from PubChem ID (Table 3). The cyclic form of glucose bound to HSA in the crystal structure was obtained from protein data bank (PDB ID: 4iw2) and served as control. The HSA target structure was retrieved from the same PDB ID (4iw2) by removing the ligands. SAL subsite of Sudlow Site I served as the active site.

### 4.10. Molecular Docking and Scoring of Ligand Poses

Molecular docking of ligands on the active site (defined above) was performed by Autodock-Vina [43]. A standard docking protocol was implemented. The binding efficiency of the glucopyranose and other ligands was also determined by the prodigy-ligand webserver [44,45].

### 4.11. Statistical Analysis

The experimental results were calculated in triplicate and expressed as mean ± standard error. Statistical analyses were performed using OriginPro 8.5 followed by *t* test. Significance was represented by *p* values < 0.05.

## 5. Conclusions

Previous researchers have demonstrated that there is a complex network of interdependencies and correlations among qualities of various natural products, which may work together to provide the overall therapeutic capabilities of different medicinal plants. The results show that the inhibition of heat-induced denaturation of protein and prevention of glycation and AGEs formation increased with an increase in garlic extract concentration. Furthermore, garlic extract exhibited significant levels of protection in protein structural stabilization against glycation. Our research significantly supports the numerous therapeutic properties of garlic and addresses the question of the interdependence of various biological activities and their antioxidant capacity. Thus, it is suggested to include garlic into any existing preventative and treatment approach for glycation-induced health complications in diabetic patients. For this purpose, a well-designed research strategy involving animal and clinical study is highly recommended for the verification of the therapeutic advantages of garlic extract.

## Figures and Tables

**Table 1:** Chemical and biological studies of aqueous garlic extract.

Preliminary Screening	Garlic Extract
Weight of dry powder	50 g
Yield	5.19%
Extract	Aqueous
Flavonoids	+
Polyphenolic compounds	+
Total phenolic compounds	21.45 ± 0.02 mg gallic acid equivalent/g dry weight of extract
Total flavonoid content	16.58 ± 0.03 mg quercetin equivalent/g dry weight of the extract

+ sign indicates presence of flavonoids and polyphenolic compounds.

**Table 2:** Secondary structure composition of N-HSA, G-HSA, and G-HSA incubated with different concentrations of garlic extract (0–100 µg/mL).

Conformation	N-HSA	G-HSA	G-HSA with Garlic Extracts (μg/mL)								
	-		0.78	1.56	3.12	6.25	12.5	25	50	100	AG
α-helix	42.7 ± 0.6	38.2 ± 0.3 (−10.5%)	38.2 ± 0.3 (−10.5%)	38.3 ± 0.3 (−10.3%)	38.4 ± 0.3 (−10.0%)	38.8 ± 0.3 (−9.1%)	39.8 ± 0.3 * (−6.8%)	40.5 ± 0.3 * (−5.2%)	40.9 ± 0.3 ** (−4.2%)	41.5 ± 0.3 *** (−2.8%)	40.8 ± 0.3 ** (−4.4%)
β-sheet	26.2 ± 0.5	30.1 ± 0.2 (+14.9%)	30.1 ± 0.2 (+14.9%)	30.0 ± 0.2 (+14.5%)	29.9 ± 0.2 (+14.1%)	29.6 ± 0.2 (+13.0%)	28.8 ± 0.2 * (+9.9%)	28.1 ± 0.2 ** (+7.3%)	27.9 ± 0.2 ** (+6.5%)	27.4 ± 0.2 *** (+4.6%)	27.8 ± 0.2 ** (+6.1%)
β-turn	18.5 ± 0.2	19.4 ± 0.3 (+4.9%)	19.4 ± 0.3 (+4.9%)	19.4 ± 0.3 (+4.9%)	19.4 ± 0.3 (+4.9%)	19.2 ± 0.3 (+4.3%)	19.1 ± 0.3 * (+3.2%)	19.0 ± 0.3 ** (+2.7%)	18.8 ± 0.3 ** (+1.6%)	18.6 ± 0.3 ** (+0.5%)	19.0 ± 0.3 ** (+2.7%)
Random coil	12.6 ± 0.5	12.3 ± 0.4 (−2.4%)	12.3 ± 0.4 (−2.4%)	12.3 ± 0.4 (−2.4%)	12.3 ± 0.4 (−2.4%)	12.3 ± 0.4 (−2.4%)	12.3 ± 0.4 (−2.4%)	12.4 ± 0.4 * (−1.6%)	12.4 ± 0.4 * (−1.6%)	12.5 ± 0.4 *** (−0.8%)	12.4 ± 0.4 * (−1.6%)

The values are in percentage. Each sample was read in triplicate. Data are mean ± standard deviation. * *p* < 0.05, ** *p* < 0.01, and *** *p* < 0.001 vs. control (N-HSA). Values in parentheses represent the percentage change in the secondary structure from N-HSA. Percentage decrease and increase are denoted by “–” and “+” signs. Different GE concentrations were used in μg/mL. The 5 mm of AG was used as negative control. The *t* test was adopted for the comparison between the groups.

**Table 3:** Ligands. Common name refers to the compound name used in the study.

Ligand/Inhibitor Name (Common Name)	IUPAC Name, Mol Formula, and PubChem ID	Binding Affinity Kcal/Mol	Number of Hydrogen Bonds	Other Interactions *
Glucose (Cyclic form)	(3R,4S,5S,6R)-6-(hydroxymethyl)oxane-2,3,4,5-tetrol Mol formula: C_6_H_12_O_6_ PubChem ID: 5793	−6.2	6 (3 × Arg257, 1 × Arg221, 1 × Tyr150, 1 × His242)	1 (1 × Lys199) Leu238 and Ala291 (Hydrophobic interactions)
Catechin	(2S,3R)-2-(3,4-dihydroxyphenyl)-3,4-dihydro-2H-chromene-3,5,7-triol Mol formula: C_15_H_14_O_6_ PubChem ID: 73160	−6.7	1 (1 × His 288)	13 (1 × Arg 257, 1 × His 288, 1 × Glc 602, 1 × Ala 291, 1 × Tyr 150, 1 × Glu 153, 1 × Glu 292, 1 × Lys 195, 1 × Ala 191, 1 × Phe 157, 1 × Ser 192, 1 × Lys 436, 1 × Glu 188 Carbon H Bond)
Caffeic Acid	(E)-3-(3,4-dihydroxyphenyl) prop-2-enoic acid Mol formula: C_9_H_8_O_4_ PubChem ID:689043	−6.6	3 (1 × Arg 222, 2 × Ser 192)	12 (1 × Arg 218, 1 × Po 4603, 1 × Glc 602, 1 × Leu 238, 1 × Tyr 150, 1 × Lys 195, 1 × Glu 153, 1 × Ala 291, 1 × His 288, 1 × Phe 157,1 × Gln 196, 1 × Lys 199)
Gallic Acid	3,4,5-trihydroxybenzoic acid Mol formula: C_7_H_6_O_5_ PubChem ID: 370	−6.4	4 (2 × Lys 199, 1 × Gln 196, 1 × Glu 153)	10 (1 × His 242, 1 × Gln 196, 1 × Ser 192, 1 × Lys 195, 1 × Tyr 150, 1 × Ala 291, 1 × Phe 157, 1 × His 288, 1 × Glu 292, 1 × Arg 257)
m-Coumaric Acid	E)-3-(3-hydroxyphenyl)prop-2-enoic acid Mol formula: C_9_H_8_O_3_ PubChem ID: 637541	−6.3	4 (1 × Arg 222, 1 × PO 4603, 1 × Glc 602, 1 × Ser 192)	6 (1 × Arg 222, 1 × PO 4603, 1 × Glc 602, 1 × Ser 192, 1 × Gln 196 Unfavorable donor–donor, 1 × Gln 196 Unfavorable donor–donor)
Quercetin	2-(3,4-dihydroxyphenyl)-3,5,7-trihydroxychromen-4-one Mol formula: C_15_H_10_O_7_ PubChem ID: 5280343	−8.1	4 (1 × Ser 192, 1 × Lys 199, 1 × Arg 257, 1 × Arg 222)	17 (1 × Lys 195, 1 × Lys 199, 1 × Gln 196, 1 × His 242, 1 × Tyr 150, 3 × Ala 291, 2 × Leu 238, 1 × Leu 260, 1 × Ala 261, 1 × Ser 287, 1 × Ile 290, 1 × Leu 219,1 × Arg 222, 1 × Glu 153)
Pyrogallol	benzene-1,2,3-triol Mol formula: C_6_H_6_O_3_ PubChem ID: 1057	−5.4	3 (2 × Ser 192, 1 × Gln 196)	8 (1 × Tyr 150, 1 × Glu 153, 1 × Phe 157, 1 × Lys 199, 1 × Ala 291, 1 × Lys 195, 1 × His 242, 1 × Arg 257)
Dihydroxybenzoic acid	2,3-dihydroxybenzoic acid Mol formula: C_7_H_6_O_4_ PubChem ID: 19	−6.2	3 (1 × Arg 222, 1 × Ser 287, 1 × Arg 257)	9 (1 × Leu 260, 1 × Ile 290, 1 × Leu 238, 1 × Ala 291, 1 × Arg 257, 1 × Leu 219, 1 × Ile 264, 1 × Ala 261, 1 × Tyr 150)

* Van der Waals, polar, pi–pi interactions, carbon–hydrogen bonds, pi–sigma, pi–alky, etc.

## Data Availability

The data presented in this study are available on request from the corresponding author.

## Sample Availability

Samples of the compounds are not available from the authors.
